# Diagnostic and Therapeutic Utility of Positive Intravascular Catheter Tip Cultures

**DOI:** 10.1128/spectrum.04022-22

**Published:** 2022-11-10

**Authors:** Patrizia Ulrich, Alexander J. Lepak, Derrick J. Chen

**Affiliations:** a UW Health, Madison, Wisconsin, USA; b University of Wisconsin-Madison, School of Medicine and Public Health, Department of Medicine, Infectious Disease Division, Madison, Wisconsin, USA; c University of Wisconsin-Madison, School of Medicine and Public Health, Department of Pathology and Laboratory Medicine, Madison, Wisconsin, USA; Johns Hopkins Hospital

**Keywords:** catheter culture, central line-associated bloodstream infection, catheter-related bloodstream infection, catheter, differential time to positivity

## Abstract

This study evaluated the performance and clinical utility of performing intravascular catheter tip cultures (CTC). A retrospective chart review was conducted over a 2.5 year period on all patients who demonstrated growth of at least one organism on CTC. There were a total of 391 CTC performed. 88 (23%) grew at least one organism, while 303 (77%) had no growth. Of the positive CTC, 81 (92%) had blood cultures (BC) collected within 14 days, whereas 7 (8%) did not. Of the positive CTC with BC, 67 (83%) were BC-positive, whereas 14 (17%) were negative. For cases with growth on both CTC and BC, the organisms identified were concordant for 46 (69%) cases and discordant for 21 (31%). Of the concordant cases, 43 (93%) were clinically considered to be bacterial bloodstream infections that were secondary to a catheter infection. For all of the positive CTC cases total, there was no change in the antibiotics or management, with the exceptions of 2 out of 88 (2%) cases. Catheters were removed and cultured for an average of 38.6 h (range: −98 to 288 h) after positive BC results were available. Most CTC are negative, and for the CTC that are positive, most are concordant with BC results. CTC results are generally only available several days after positive BC results are known. The CTC results did not alter the antibiotic therapy or management, with the exceptions of rare cases. As such, this study concludes that CTC do not contribute diagnostic or therapeutic value. Therefore, current guidelines by the Infectious Diseases Society of America on catheter-related bloodstream infection diagnosis should be revised to exclude CTC collection.

**IMPORTANCE** In patients with intravascular catheters who are febrile or have positive blood cultures and no other obvious sources of infection, catheter tip cultures are often obtained to evaluate potential catheter-related bloodstream infections. However, previous studies reported that the management of catheter-related bloodstream infection cases is entirely based on blood culture growth and susceptibilities and that catheter tip cultures have low diagnostic positive predictive value. Our study represents the largest contemporary evaluation that includes chart reviews on all positive catheter tip culture cases. We found that positive cultures led to no changes in antibiotics or management, except for in two cases. Furthermore, 92% of positive catheter tip cultures were associated with blood culture collections, and catheter cultures were generally available only several days after the blood culture results were known. Thus, our study supports the claim that positive catheter tip cultures add limited diagnostic and therapeutic value in suspected catheter-related bloodstream infections.

## INTRODUCTION

There are approximately 250,000 cases of central venous catheter-associated bloodstream infections annually in the United States. Estimates of the total annual cost of catheter-related bloodstream infections range from $296 million to $2.3 billion, and mortality ranges from 12 to 25% per episode (1).

Given that the signs and symptoms associated with catheter-related bloodstream infections, such as fever, chills, and catheter exit site erythema, are not specific or sensitive for the diagnosis of catheter-related bloodstream infections, microbiological evidence is required (2). Per the Infectious Diseases Society of America (IDSA), a definitive diagnosis of a catheter-related bloodstream infection is made when concordant growth on blood culture (BC) and catheter tip culture (CTC) is seen. Additionally, >15 colony-forming units (CFU) are required for catheter tip cultures performed via the semiquantitative method. Alternative and catheter-sparing methods by which to define catheter-related bloodstream infections include obtaining one BC drawn from the catheter hub and another BC drawn peripherally, and this is followed by a subsequent comparison of these for a simultaneous quantitative blood culture ratio of >5:1 or a differential time to positivity that is ≥2 h (3).

In patients with intravascular catheters who are febrile or have positive BC and no other obvious sources of infection, catheters are often suspected as the underlying source. As such, CTC are often obtained to evaluate catheter-related bloodstream infections. However, a 1992 study by Widmer et al. demonstrated that only 4% of 157 CTC effectively influenced clinical decisions (4). More recent studies demonstrated that the management of catheter-related bloodstream infection cases is entirely based on BC growth and susceptibilities and that CTC have a positive predictive value for catheter-related bloodstream infections in only 11% of cases (5, 6). Given the unclear impact of the CTC results, our study sought to determine what clinically relevant information is contributed by positive CTC in our health care system.

## RESULTS

**FIG 1:**
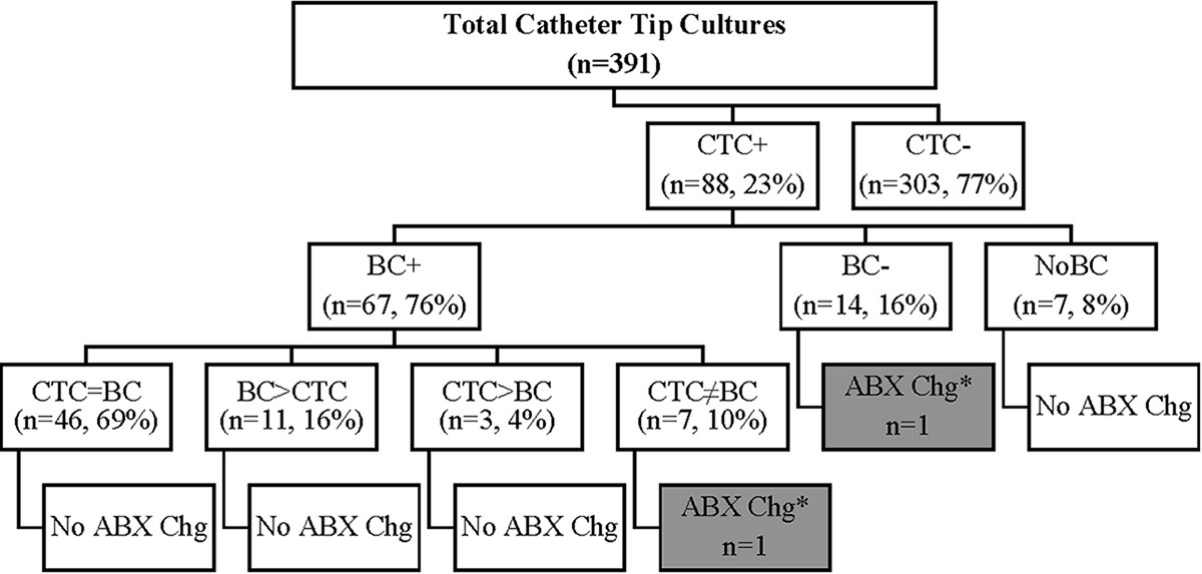
Classification of catheter tip and blood culture results, according to the presence of organism growth and the changes to antibiotic management. CTC, catheter tip culture; BC, blood culture; + or −, growth or no growth. NoBC, no BC obtained within 14 days of CTC; CTC = BC, CTC growth that is the same as the BC growth; BC > CTC, BC grew an additional organism, compared to CTC; CTC > BC: CTC grew an additional organism, compared to BC; CTC ≠ BC, CTC and BC grew different organisms. ABX Chg, change in antibiotics. An asterisk indicates a case in which antibiotic changes were identified: vancomycin was added for a localized Staphylococcus epidermidis skin infection in 1 CTC ≠ BC case, and vancomycin was added for CTC Staphylococcus aureus growth in 1 CTC^+^/BC^−^ case.

**FIG 2:**
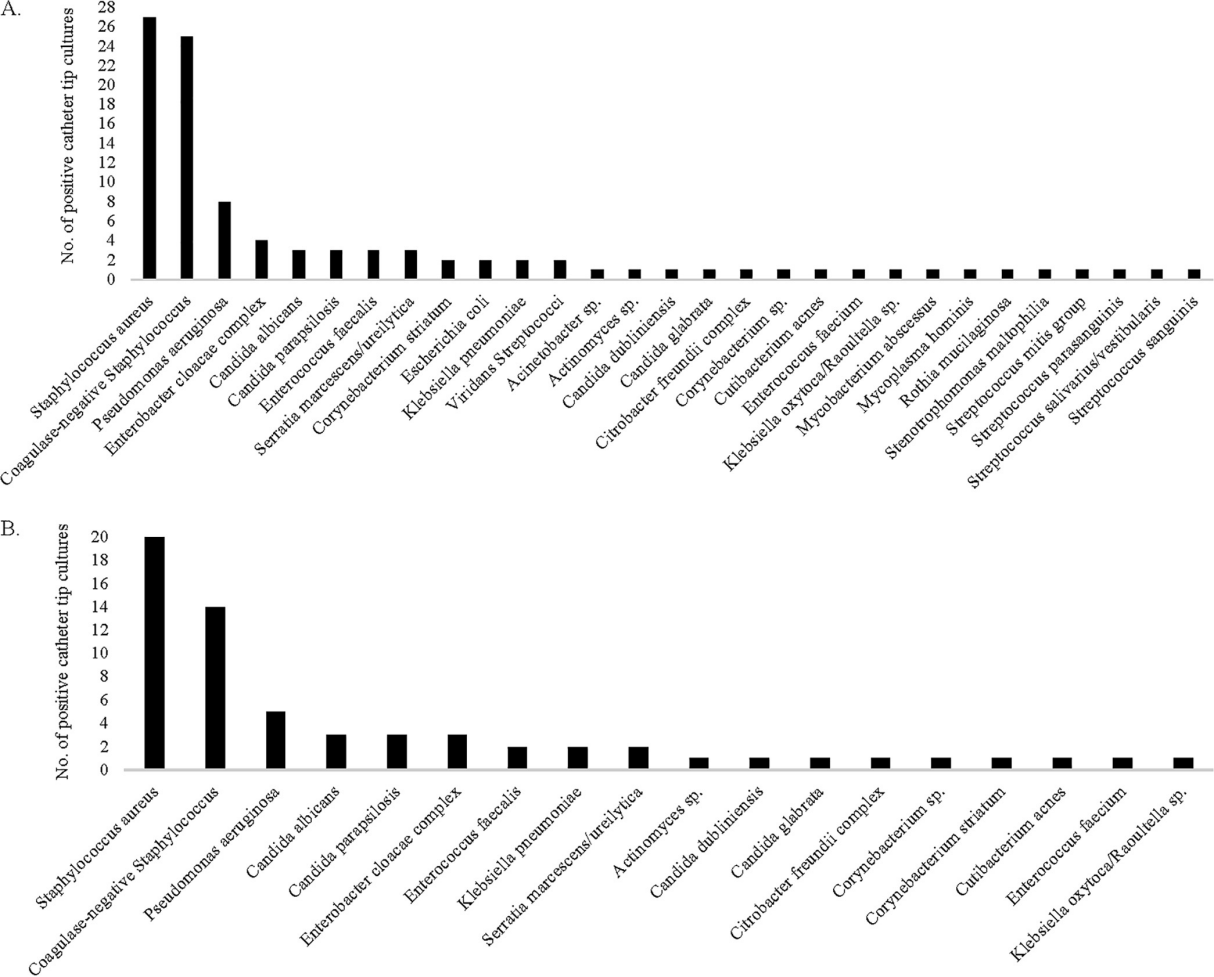
Identification of organisms recovered from catheter tip cultures. (A) All positive catheter tip cultures (*n* = 101 isolates; 10 out of 88 catheter tip cultures were polymicrobial). (B) Organisms identified from catheter tip cultures for cases in which catheters were the source of a bloodstream-infection or a fever (*n* = 63).

There were a total of 391 CTC performed. 88 (23%) grew at least one organism, whereas 303 (77%) had no growth (Fig. 1). For the positive CTC cases, the mean patient age was 49 years (range:1 to 81 years), 101 organisms were identified, and 10 cases were polymicrobial. There were 27 (27%) S. aureus recovered, 25 (25%) coagulase-negative Staphylococcus (*n* = 16 Staphylococcus epidermidis), 8 (8%) *Candida* sp. (*n* = 3 C. albicans), 8 (8%) Pseudomonas aeruginosa, 6 (6%) Streptococcus sp., 4 (4%) Enterobacter cloacae complex, 4 (4%) *Enterococcus*, and 19 (19%) instances of 12 other species (Fig. 2A). The types of catheters from which positive CTC were obtained are summarized in Table 1.

**TABLE 1:** Catheter types from which positive catheter tip cultures were obtained (*n* = 88)

Type	Frequency (%)
Port catheter	36 (41)
Hickman catheter	21 (24)
Peripherally inserted central catheter	15 (17)
Internal jugular vein catheter	14 (16)
Dialysis catheter	1 (1)
Swan-Ganz catheter	1 (1)

Of the positive CTC, 81 (92%) had blood cultures collected within 14 days and 7 (8%) did not. 67 (83%) of the BC were positive, and 14 (17%) were negative (Fig. 1). The catheters were removed and cultured on average 38.6 h after positive BC results became available (*n* = 65; 2 cases excluded due to a lack of data), ranging from CTC being obtained 98 h before BC collection to 288 h after BC collection.

For cases with both positive CTC and positive BC results, the organisms identified were concordant (i.e., identical organisms were identified on CTC and on BC) for 46 (69%) of the cases and discordant for 21 (31%); the discordant cases either were due to additional organisms being found in BC for 11 cases or in CTC for 3 cases or were due to completely different organisms being recovered in CTC versus BC for 7 cases (Fig. 1).

**FIG 3:**
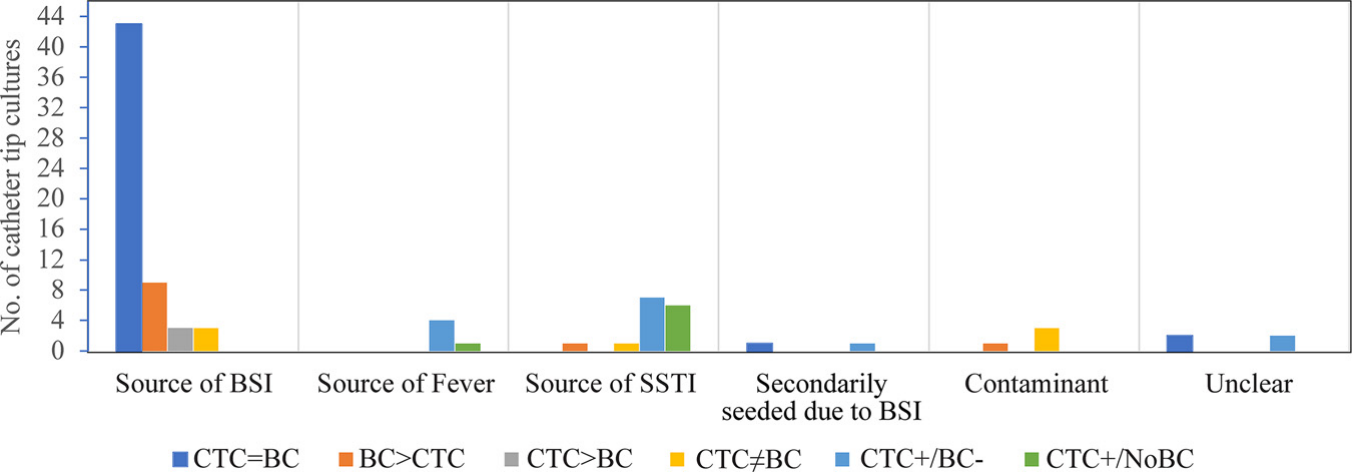
Clinical interpretation of positive catheter tip cultures (*n* = 88), classified according to blood culture concordance. SSTI, skin and soft tissue infection; BSI, bloodstream infection; CTC, catheter tip culture; BC, blood culture; CTC = BC, catheter tip culture growth that is same as the blood culture growth; BC > CTC, the blood culture grew an additional organism, compared to the catheter tip culture; CTC > BC, the catheter tip culture grew an additional organism, compared to the blood culture; CTC ≠ BC, the catheter tip culture and the blood culture grew different organisms. CTC^+^/BC^−^, growth on catheter tip culture but no growth on blood cultures; CTC^+^/NoBC, no blood culture obtained within 14 days of CTC.

Catheters were considered to be the source of the bloodstream infections in 58 (66%) of the positive CTC cases. 43 of these had concordant growth on CTC and BC, and the remaining 15 were discordant cases. Catheters were considered to be the source of fever in 5 (6%) of the positive CTC cases. Of these, 4 had a positive CTC but a negative BC, and 1 had a positive CTC but no BC. Catheters were the sources of localized skin and soft tissue infections at the catheter exit sites for 15 (17%) of the positive CTC cases. Of these, 7 had a positive CTC but a negative BC, 6 had a positive CTC with no BC, 1 had an additional organism identified on BC, and 1 case grew entirely distinct organisms on CTC and BC. Catheter growth was determined to be a contaminant in 4 of the positive CTC cases. Of these, 3 grew entirely distinct organisms on CTC and BC, and 1 grew an additional organism on BC. The secondary seeding of the catheter and an unclear clinical determination of positive CTC culture were rare (CTC results are summarized in Fig. 3). Out of the 63 bloodstream infection-or fever-associated positive CTC cases, 20 (32%) were S. aureus, 14 (22%) were coagulase-negative Staphylococcus, and 9 (14%) were *Candida* sp. (Fig. 2B).

Out of the 67 CTC with positive BC, 31 had centrally drawn and peripherally drawn BC specimens available for the evaluation of the differential time to positivity. These differential time to positivity results became available an average of 40.2 h (range: −5 to 288 h) before the catheters were removed and cultured. 18 (58%) of the 31 available cases had a differential time to positivity of ≥2 h (i.e., centrally drawn BC results were positive ≥2 h before those of peripherally drawn BC). In 17 out of 18 cases with a differential time to positivity of ≥2 h, the catheters were considered to be the sources of the bacteremia.

The incidence of changes to antibiotic management (defined as the initiation of new antibiotics, antibiotic dosage changes, or antibiotic class switches) are summarized in Fig. 1. For all 88 positive CTC cases, regardless of BC status, there were only 2 cases in which antibiotic management was changed: 1 case in which CTC and BC grew entirely separate organisms and vancomycin was added for a S. epidermidis localized skin infection and 1 case with positive CTC but negative BC, in which vancomycin was added for S. aureus. Antibiotics were started an average of 5.4 days (range: 0 to 18 days) before the CTC results became available.

## DISCUSSION

Catheter-related bloodstream infections are one of the leading causes of nosocomial infections and are associated with significant patient mortality and health care costs (1). Although culturing catheter tips has been traditionally used to confirm suspected catheter-related bloodstream infection cases, the overall clinical utility of these cultures has been questioned.

In our study, we found that out of 391 intravascular CTC, most (77%) of the CTC showed no growth. Of the CTC that were positive, most (69%) demonstrated concordant growth on BC when it was performed. We found that the choice of antibiotic was guided by the BC results, rather than CTC results, and the BC susceptibilities in 66 out of 67 cases that demonstrated both positive CTC and positive BC. For the one case with growth on both CTC and BC, in which antimicrobial therapy changed based on positive CTC results, CTC growth was used to guide therapy for a localized skin and soft tissue infection at the catheter exit site. Out of the cases with positive CTC but negative BC results, we found one instance in which S. aureus CTC growth led to the subsequent addition of vancomycin in a patient with a recent history of S. aureus catheter-related bloodstream infection. Initiating antimicrobial therapy in this circumstance was in concordance with previous literature and with the current IDSA recommendation (3, 7). Our finding that CTC growth impacted antibiotic therapy in only 2 out of 88 cases is in concordance with the findings of Widmer et al., who evaluated 157 CTC results and identified a clinical impact in only 4% of patients, and, even among those impacted, the authors questioned the appropriateness of the changes. Hung et al., who evaluated 68 suspected catheter-related bloodstream infection cases, similarly demonstrated that CTC had no impact on antimicrobial therapy in catheter-related bloodstream infection cases (5). As such, the impact of positive CTC results on therapeutic decisions appears to be limited, and CTC should not be collected for the management of catheter-related bloodstream infections. The low incidence of antibiotic changes identified in our and other studies may be explained by the findings that CTC results were only available several days after positive BC results were known and that antibiotics had already been started an average of >5 days before. Cooper et al. conducted chart reviews on 52 tunneled hemodialysis CTC and similarly found that in almost all of the cases, the BC results were available at least 24 h before the CTC results were reported (8).

Chart reviews revealed that the majority of positive CTC were initially collected due to a clinical suspicion for a catheter-related bloodstream infection, based on patient presentations. Out of cases with concordant CTC and BC growth in our study, most (93%) of the catheters were clinically considered to be the underlying source of the patient’s bloodstream infection. In cases in which centrally and peripherally drawn blood specimens were collected, the data of the differential time to positivity, which informed whether catheters were the underlying source of the bloodstream infections, were available an average of 40.2 h before the CTC were collected. Overall, thes superior utility of the differential time to positivity over CTC is evidenced by a meta-analysis by Safdar et al., which found that catheter-sparing diagnostic methods, including the differential time to positivity, have equal or greater sensitivity and specificity to those of CTC for diagnosing catheter-related bloodstream infections (9). In a 2015 letter to *Clinical Infectious Diseases*, Lance Peterson reiterated the inadequacy and the potential risk of CTC, based on its unreliable and generally poor positive predictive value for catheter-related bloodstream infections (10). Ferreira et al. compared the clinical utility of catheter-drawn blood versus CTC for neonatal catheter-related bloodstream infection cases and found no difference in patient outcomes between the two methods (11). Thus, although CTC results can contribute to the diagnosis of catheter-associated bacteremia, the differential time to positivity is often available before the CTC results. Therefore, not only do CTC results have little to no impact on patient management, but their diagnostic utility for catheter-related bloodstream infections is limited due to the use of the differential time to positivity. In fact, Lei et al. found a sharp decline in the collections of vascular tip cultures between 2009 and 2014, which the authors partially attributed to emerging evidence that fails to support the diagnostic and therapeutic value of CTC (12).

Further supporting catheter-sparing diagnostic methods is that catheter removal may not always be required: in uncomplicated, long-term CVC or port related bacteremia due to pathogens other than S. aureus, P. aeruginosa, *Bacillus* sp., *Micrococcus* sp., *Propionibacterium* sp., fungi, or mycobacteria, catheter-sparing treatments may be employed according to the Infectious Diseases Society of America guidelines (3). Our study found that 46% of the isolates identified from fever-associated or bloodstream infection-associated positive CTC would fall into this catheter-sparing treatment category.

There are limitations to our study. We did not evaluate the impact of negative CTC. Although Cooper et al.’s study on tunneled hemodialysis CTC reported that none of the 108 negative CTC resulted in the discontinuation of antibiotics, no studies have specifically evaluated the clinical impacts of negative CTC results. Negative CTC results could theoretically provide other clinically valuable information, based on the absence of CTC growth, and affect the reevaluation and diagnosis of other underlying bloodstream infection sources. Additionally, our study was not designed to evaluate the impact on line management, which is an important consideration in many patient populations. CTC results, both positive and negative, may impact the need for temporary lines, line holidays, and timing of future line placement for the patient. These are important considerations for future studies.

In summary, our study is the largest contemporary evaluation that included chart reviews on all positive CTC cases, regardless of the reason for collection. The validity and accuracy of our findings is supported by similar results reported in literature. Additionally, Flynn et al. found that the cost and technician hours for 1,391 CTC were $75,300 and 600 h, respectively (13). Taken together, these studies collectively support the claim that positive CTC results add limited value to the management of catheter-related bloodstream infections, have a limited impact on patient mortality, have a low sensitivity for catheter-related bloodstream infection diagnoses, and are not cost-effective. As such, the current Infectious Diseases Society of America guidelines on the diagnostic criteria for catheter-related bloodstream infections should be revisited to exclude the practice of CTC collection.

## MATERIALS AND METHODS

A retrospective cohort study was conducted over a 2.5 year (May 2019 to December 2021) period and used the electronic health record reviews of UW Health University Hospital patients who demonstrated growth of at least one organism on intravascular CTC. CTC was performed via the semiquantitative roll plate method with a 3-day incubation period. Staphylococcus aureus, Staphylococcus lugdunensis, Streptococcus pyogenes, *Enterococcus* spp., and Gram-negative rods were reported in any quantity, and other organisms were reported only when ≥15 colony-forming units (CFU). Charts of positive CTC cases were evaluated for the presence of BC obtained within 2 weeks before or after CTC collection. If BC were available, cases were evaluated for concordance between BC and CTC growth and the differential time to positivity. Cases with positive CTC were further evaluated for other nonblood specimens that were cultured within 2 weeks of the CTC and for the time of antibiotic administration, relative to CTC collection. Lastly, the clinical significance of CTC results, as determined by the primary or infectious disease physicians involved in the care, was recorded, as were the changes in antibiotics or management implemented based on the return of positive CTC results.

This project was conducted as part of a quality improvement project. Thus, institutional review board (IRB) approval was not required.

### Data availability.

Data are not shared on the public data repository, given that protected health information is included in the data set and that patient confidentiality must be protected. Select data may be available via requests sent to Derrick Chen, the medical director of the clinical microbiology laboratory: dchen@uwhealth.org.
